# Polymorphisms in IL-1β, vitamin D receptor *Fok*1, and Toll-like receptor 2 are associated with extrapulmonary tuberculosis

**DOI:** 10.1186/1471-2350-11-37

**Published:** 2010-03-02

**Authors:** Alison A Motsinger-Reif, Paulo RZ Antas, Noffisat O Oki, Shawn Levy, Steven M Holland, Timothy R Sterling

**Affiliations:** 1 Bioinformatics Research Center, Department of Statistics, North Carolina State University, Raleigh, NC, USA; 2 Division of Infectious Diseases, Department of Medicine, Vanderbilt University School of Medicine, Nashville, TN, USA; 3 Department of Biomedical Informatics, Vanderbilt University School of Medicine, Nashville, TN, USA; 4 Laboratory of Clinical Infectious Diseases, National Institutes of Health, Bethesda, MD, USA; 5 Center for Health Services Research, Department of Medicine, Vanderbilt University School of Medicine, Nashville, TN, USA

## Abstract

**Background:**

Human genetic variants may affect tuberculosis susceptibility, but the immunologic correlates of the genetic variants identified are often unclear.

**Methods:**

We conducted a pilot case-control study to identify genetic variants associated with extrapulmonary tuberculosis in patients with previously characterized immune defects: low CD4+ lymphocytes and low unstimulated cytokine production. Two genetic association approaches were used: 1) variants previously associated with tuberculosis risk; 2) single nucleotide polymorphisms (SNPs) in candidate genes involved in tuberculosis pathogenesis. Single locus association tests and multifactor dimensionality reduction (MDR) assessed main effects and multi-locus interactions.

**Results:**

There were 24 extrapulmonary tuberculosis cases (18 black), 24 pulmonary tuberculosis controls (19 black) and 57 PPD+ controls (49 black). In approach 1, 22 SNPs and 3 microsatellites were assessed. In single locus association tests, interleukin (IL)-1β +3953 C/T was associated with extrapulmonary tuberculosis compared to PPD+ controls (P = 0.049). Among the sub-set of patients who were black, genotype frequencies of the vitamin D receptor (VDR) *Fok*1 A/G SNP were significantly different in extrapulmonary vs. pulmonary TB patients (P = 0.018). In MDR analysis, the toll-like receptor (TLR) 2 microsatellite had 76% prediction accuracy for extrapulmonary tuberculosis in blacks (P = 0.002). In approach 2, 613 SNPs in 26 genes were assessed. None were associated with extrapulmonary tuberculosis.

**Conclusions:**

In this pilot study among extrapulmonary tuberculosis patients with well-characterized immune defects, genetic variants in IL-1β, VDR *Fok*1, and TLR2 were associated with an increased risk of extrapulmonary disease. Additional studies of the underlying mechanism of these genetic variants are warranted.

## Background

A possible genetic predisposition to tuberculosis has been suggested in several studies,[1-3] but the functional immunologic correlate of the genetic polymorphisms identified is often unclear[4]. We have sought to first identify immunologic defects that may predispose to tuberculosis, then assess for genetic polymorphisms associated with these immunologic defects. Because there is evidence that extrapulmonary tuberculosis is the result of an underlying immune defect, [5] we have focused our search on persons with prior extrapulmonary disease. In previous studies we noted that HIV-seronegative adults with prior extrapulmonary tuberculosis had lower levels of CD4+ lymphocytes, unstimulated cytokine production, and tumor necrosis factor (TNF)-α production in response to lipopolysaccharide (LPS) or LPS + interferon (IFN)-γ than persons with prior pulmonary tuberculosis or latent *M. tuberculosis *infection[6,7].

In the current study we have combined two previous study populations in which cytokine responses were well-characterized, [6,7] to identify genetic polymorphisms associated with extrapulmonary tuberculosis, and by extension, the associated immunologic abnormalities. We make use of a candidate gene approach utilizing single nucleotide polymorphisms (SNPs) and microsatellites previously reported to be associated with tuberculosis, and we also include SNPs in candidate genes that are hypothesized to play a role in tuberculosis pathogenesis.

Because several loci could contribute to the phenotype expressed in complex diseases such as tuberculosis, it is important to search for gene-gene interactions (epistasis), which may give a more accurate prediction of disease risk. Such interactions are difficult to detect using traditional statistical methods such as single locus association tests because those tests were not developed to detect purely interactive effects. Such tests identify genes with main effects and then follow-up analyses assess for interactions between genes that exhibit a main effect. New statistical and computational methods that have better power to detect interactions, including those without main effects, in relatively small sample sizes, are required. Multifactor dimensionality reduction (MDR) analysis is a novel method developed to address this need[8]. In the current study both single locus association tests and MDR analysis were used to detect potential single or multi-locus interactions that predict extrapulmonary tuberculosis.

## Results

### Clinical and Demographic Characteristics

There were 24 extrapulmonary tuberculosis cases (18 black), 24 pulmonary tuberculosis controls (19 black), and 57 controls (49 black) with latent *M. tuberculosis *infection. The clinical and demographic characteristics of the study population are in Table 1. There were no significant differences between cases and controls according to age, sex, or race. However, pulmonary controls had lower median body mass index (BMI) at the time of study than either extrapulmonary tuberculosis cases or controls with latent *M. tuberculosis *infection. Extrapulmonary tuberculosis cases had lower median CD4+ lymphocytes at time of study than controls. The sites of disease among extrapulmonary cases included lymphatic (n = 9), pleural (n = 4), bone/joint (n = 4), pericardial (n = 2), genito-urinary (n = 2), miliary/meningeal (n = 1), gastrointestinal (n = 1), and laryngeal (n = 1).

**Table 1:** Clinical and Demographic Characteristics of the Study Population

Characteristic	Extrapulmonary TB (n = 24)	Pulmonary TB (n = 24)	PPD+ (n = 57)	P value^a^
Age at study entry (years)	48.4 (43.6-79.4)	43.6 (40.3-53.9)	45.7 (38.8-53.3)	0.18
# Male Sex (%)	16 (67)	14 (58)	36 (63)	0.84
# Black Race (%)	18 (75)	19 (79)	49 (86)	0.46
# White Race (%)	4 (17)	4 (17)	8 (14)	0.93
# Asian Race (%)	2 (9)	1 (4)	0 (0)	0.11
BMI at study entry (kg/m^2^)	25.5 (23.0-29.6)	20.6 (18.2-24.0)	25.9 (22.8-30.5)	< 0.001
CD4 count at study entry	701 (565-1015)	814 (594-938)	879 (703-1114)	0.05

Data are medians (inter-quartile range; IQR) except as noted.

^a ^Kruskal-Wallis test for continuous variables; Chi-square test for categorical variables.

### SNPs Associated with Tuberculosis in Previous Studies

Of the 27 SNPs and 3 microsatellites previously reported in the literature as being associated with tuberculosis (Table 2), 25 SNPs and all 3 microsatellites were assessed by PCR-based SNP genotyping. The IL-1β -511 (C→T) and vitamin D receptor (VDR) *Bsm*1 SNPs were on the GeneChip^® ^Human Mapping 50K Xba Array. Of the 30 polymorphisms tested, 5 SNPs (rs 17235416, rs1800450, rs5743708, rs6265786, rs2393799) were unable to be genotyped after quality control and were therefore excluded. Only markers with < 10% missing data were considered for analysis. No imputation of missing values was performed. For univariate analyses, missing values were removed for analysis; for the MDR analysis, missing observations were ignored, such that attribute construction and accuracy calculations were only performed based on observed values.

**Table 2:** Polymorphisms Tested that had Previously Been Reported as Associated with Tuberculosis

Gene	SNP	rs number	References
NRAMP1(Slc11a1)	5'(GT)_n _microsatellite	------------	[1,22-26]
	INT4 (469+14G/C)	rs3731865	[1,26]
	D543N	rs17235409	[1,27,24,28,26]
	3'UTR (1729+55del4)	rs17235416	[1,27,28,26]
	274C/T	rs2276631	[29]
SP110	sp110 intron 6	rs2114592	[30]
	sp110 exon 11	rs3948464	[30]
IL-12/23/IFN-γ Pathway	+874A/T	rs2430561	[31-35]
	-1616G	rs2069705	[36]
	+3234T	rs2069718	[36]
	IFNGR1 -56C/T	rs2234711	[36]
IL-1 and IL-1 RA	IL-1α -899 C/T	rs1800587	[37]
	IL-1β +3953 C/T	rs1143634	[38]
	IL-1β -511 C/T	rs16944	[39]
	IL-1RA microsatellite	-------------	[40]
IL-10	-1082 G/A	rs1800896	[41,34,42]
	-592 A/C	rs1800872	[43]
MBL	Codon 52 C/T (allele D)	rs5030737	[44]
	Codon 54 A/G (allele B)	rs1800450	[44]
	Codon 57 A/G (allele C)	rs1800451	[44]
VDR	*Taq*1 C/T	rs731236	[11,2,45]
	*Fok*1 A/G	rs10735810	[11,28]
	*Bsm*1	rs154410	[45]
MCP-1	-2518A/G	rs1024611	[46]
TIRAP	C558T	rs7932766	[47]
TLR2	GT repeat intII microsatellite	--------------	[18]
	Arg753Gln	rs5743708	[48]
	Arg677Trp	rs6265786	[49]
P2X7	-762T/C	rs2393799	[50]
	1513A/C	rs3751143	[51]

Polymorphisms were reported in the literature prior to November 15, 2006. There were 27 single nucleotide polymorphisms (SNPs) and three microsatellites. The status of each polymorphism tested in this study is provided.

Abbreviations (names of genes):

NRAMP-1 natural resistance-associated macrophage protein-1

SP110 speckled 110, nuclear hormone receptor co-activator

IL-12/23 interleukin-12/23

IFN-γ interferon-gamma

IFNGR1 interferon-gamma receptor 1

VDR vitamin D receptor

IL-1 interleukin-1

MCP-1 monocyte chemoattractant protein-1

IL-1 RA interleukin-1 receptor antagonist

TIRAP toll-interleukin 1 receptor adapter protein

IL-10 interleukin-10

TLR2 toll-like receptor 2

MBL mannose binding lectin

P2X7 purinoreceptor

In single locus association testing of each polymorphism among all study participants, the IL-1β +3953 (C→T) SNP was associated with any tuberculosis (P = 0.044) and extrapulmonary tuberculosis (P = 0.049) compared to PPD+ controls (Table 3). Among black patients, the *Fok1 *SNP in the VDR gene was associated with extrapulmonary tuberculosis compared to pulmonary tuberculosis controls (P = 0.018).

**Table 3:** Of the Polymorphisms Tested that had Previously Been Reported as Associated with Tuberculosis (Table 2), the SNPs Associated with Tuberculosis in this Study

Comparator groups	SNPs	P-value
*Single Locus Association Tests*		
*All patients*		
Any TB vs. PPD+	IL-1beta +3953	0.044
ExtTB vs. PPD+	IL-1beta +3953	0.049
ExtTB vs. PulmTB	none	---
*Black patients*		
Any TB vs. PPD+	none	---
ExtTB vs. PPD+	none	---
ExtTB vs. PulmTB	VDR Fok1	0.018
*Multifactor Dimensionality Reduction Analysis*		
*All patients*		
Any TB vs. PPD+	TLR2 microsatellite	0.038#
ExtTB vs. PPD+	none	---
ExtTB vs. PulmTB	none	---
*Black patients*		
Any TB vs. PPD+	TLR2 microsatellite	0.047*
ExtTB vs. PPD+	TLR2 microsatellite	0.002^
ExtTB vs. PulmTB	none	---

SNPs with P ≤ 0.05 are listed. P-values are uncorrected.

# 61% prediction accuracy

* 63% prediction accuracy

^ 76% prediction accuracy

In MDR analysis of the 25 polymorphisms among all study participants, the microsatellite in TLR2 (GT) had 61% prediction accuracy for any tuberculosis compared to controls with *M. tuberculosis *infection (P = 0.038) (Table 3). It also had 63% prediction accuracy for any tuberculosis in blacks (P = 0.047) and 76% prediction accuracy for extrapulmonary tuberculosis in blacks (P = 0.002). Results did not change when the analysis included CD4+ lymphocytes and BMI as potential covariates (results not shown).

### SNPs in Candidate Genes

There were 661 SNPs in genes presumed to be associated with tuberculosis pathogenesis (Table 4), of which 613 (93%) had genotyping efficiency > 90% (i.e., < 10% missing data) and were included in the analysis. In the MDR analysis of these 613 SNPs, an intronic SNP in TNF-α (C→T) (rs1811063) predicted tuberculosis with 62% accuracy among all patients (P = 0.05; Table 5). The combination of two SNPs in TLR4 (C→T) (rs1399431) and TNF-α (C→T) (rs7791836) predicted tuberculosis risk with 71% accuracy in blacks (P = 0.02). Results did not change when the analysis included CD4+ lymphocytes and BMI as potential covariates (results not shown). There were no SNPs associated with extrapulmonary tuberculosis compared to controls.

**Table 4:** Polymorphisms in the Affymetrix GeneChip^® ^Human Mapping 50K Xba Array in Genes Hypothesized to be Associated with Tuberculosis Pathogenesis

Gene	# SNPs	Gene	# SNPs	Gene	# SNPs
IL-4	4	GATA3	68	IFN-α	5
IL-6	11	TRAF6	3	CYLD	13
IL-8	5	HLA-DQ	5	BCL10	1
IL-10	4	HLA-DR	5	A20	28
IL-12	28	VDR	2	TAK1	7
TNF	191	NOD2/CARD15	1	STAT4	9
IFN-γ	18	NFκ b	50	IL-23	6
IL-1β	2	STAT1	3	TLR4	21
RunX	66	TGF-β	105		

The number of SNPs tested in each gene is provided. There were a total of 661 SNPs.

**Table 5:** Of the SNPs Listed in Table 4, the SNPs Associated with Tuberculosis in This Study Population

RS number	Gene	Minor Allele Frequency	Average Testing Balanced Accuracy	Cross-validation Consistency	P-value
**Any TB vs. PPD+, all patients**					
Rs1811063	TNF-α	0.121	62.11^#^	4/5	0.05
					
**Any TB vs. PPD+, black patients**					
Rs1399431 Rs7791836	TLR4 TNF-α	0.401 0.5	71.27^^^	3/5	0.02

^#^Testing Sensitivity: 0.55; Testing Specificity: 0.69

^^ ^Testing Sensitivity: 0.71; Testing Specificity: 0.72. In post-hoc logistic regression analysis using these 2 SNPs, a recessive encoding for each SNP maximized the model fit.

The above are Multifactor Dimensionality Reduction models. Only models with P ≤ 0.05 are presented here.

## Discussion

In this study population we first identified immunologic defects among persons with extrapulmonary tuberculosis, then explored possible associations with candidate genetic variants. In single locus association tests among genetic variants previously associated with tuberculosis susceptibility, IL-1β +3953 was associated with extrapulmonary disease, as well as all forms of tuberculosis, compared to persons with *M. tuberculosis *infection. Among black patients, the *Fok*1 SNP in the VDR gene distinguished extrapulmonary from pulmonary disease. The MDR analysis provides important additional results, however, because it is more powerful than traditional logistic regression analysis,[9] adjusts for multiple comparisons via permutation testing, and because the results were more consistent across the groups assessed. MDR also evaluates potential gene-gene and gene-environment interactions, which are important to investigate in complex phenotypes such as tuberculosis pathogenesis. The TLR2 microsatellite predicted extrapulmonary tuberculosis in black patients with 76% accuracy. It also predicted all forms of tuberculosis in blacks, as well as in the full study population. The prediction accuracy estimates the importance of variables in the dataset, with the intent of generalizing the model to the full population.

These results point to the importance of IL-1β +3953, VDR *Fok*1 A/G, and the TLR2 microsatellite in extrapulmonary tuberculosis risk, with the latter two being particularly significant in blacks, even in such a small sample size. This is important in light of the increased proportion of extrapulmonary tuberculosis in blacks[5]. Further studies are needed to determine if these polymorphisms could account for this epidemiological finding. This study included very few individuals of other racial backgrounds, making comparisons of genetic models between racial groups impossible. Our results are consistent with the previously noted association between IL-1β +3953 and tuberculous pleurisy, and VDR *Fok*1 and several forms of extrapulmonary tuberculosis--both noted among in Gujarati Asians living in London[10,11]. They are also consistent with the recently identified link between TLR and the innate immune response to *M. tuberculosis*: the relationship between TLR signaling, the up-regulation of the VDR, and vitamin D-mediated killing of intracellular *M. tuberculosis *via the microbial peptide cathelicidin[12]. In that study, blacks had low 25-hydroxyvitamin D levels and low cathelicidin messenger RNA induction. While immediate biological interpretation of our results is not possible, defects in *M. tuberculosis *recognition and/or subsequent intracellular signaling in the nuclear factor kappa B (NF-κB) pathway related to TLR2 polymorphisms would be consistent with the subtle innate immune defects in the extrapulmonary tuberculosis patients in this study population: low unstimulated cytokine production, decreased TNF-α production in response to LPS or LPS + IFN-γ, and low CD4+ lymphocytes[6,7,4].

The results from SNPs in genes hypothesized to play a role in tuberculosis pathogenesis showed that no SNPs were significantly associated with extrapulmonary tuberculosis. There was, however, a SNP in TNF-α that was associated with tuberculosis among all study participants. And among blacks, a combination of SNPs in TNF-α and TLR4 predicted tuberculosis risk with 71% accuracy. These SNPs cannot be interpreted as easily in light of the immunologic findings, which pertain to extrapulmonary tuberculosis. However, they suggest that similar cytokine pathways as those noted above are important in tuberculosis pathogenesis.

It was notable that persons with prior extrapulmonary tuberculosis or latent *M. tuberculosis *infection had significantly higher body mass index (BMI) than persons with pulmonary tuberculosis. If BMI after completion of treatment is comparable to BMI prior to development of disease, it would suggest that lower BMI may predispose to pulmonary tuberculosis. It should be noted that the median BMI in persons with pulmonary disease was within the normal range, whereas BMI in persons in the other two groups was high. A recent large population-based study in Hong Kong found that as BMI increased, the risk of pulmonary tuberculosis decreased, but the risk of extrapulmonary tuberculosis did not[13]. The protective effect of increased BMI on pulmonary disease in that study persisted even after controlling for confounding variables, including smoking and diabetes mellitus. The above findings suggest that risk factors for extrapulmonary tuberculosis are not ameliorated by increased BMI--which would be consistent with an immunogenetic predisposition to extrapulmonary tuberculosis. Factors related to the *M. tuberculosis *strain also play a role in extrapulmonary disease[14].

The most important limitation of the current study was the small sample size. This was driven by the intensive immunologic testing performed on specimens from each participant. Statistically significant genetic associations were indeed identified, but the sample was under-powered (with either traditional methods or MDR analysis) to detect other potential associations. Additionally, even for the positive associations detected, reliable estimates of effect size cannot be determined, due to the "winner's curse" related to the small sample size[15]. Table 6 shows the detectable effect sizes with the current sample size for the variants previously associated with tuberculosis. These calculations assume 80% power and an uncorrected alpha of 0.05%. Larger studies are needed in which candidate gene polymorphisms and associated cytokine pathways are fully evaluated in all patients, so that any genetic polymorphisms identified can be interpreted in the context of the cytokine abnormalities noted. Given this, one should consider the results of this study--particularly those from the candidate gene approach and interaction analysis--as hypothesis-generating rather than hypothesis-confirming. While MDR has greater statistical power than traditional methods, its power to detect gene-gene and gene-environment interactions is less than that to detect main effects. In searching for interactive effects, the small sample size did not allow for investigating interactions with more than two loci.

**Table 6:** Odds Ratios that Could Have Been Detected Given the Sample Size and Minor Allele Frequency in the Study Population

SNP	rs number	Minor Allele Frequency	Detectable Odds Ratio*
NRAMP1 INT4	rs3731865	0.12	4.19
NRAMP1 D543N	rs17235409	0.07	5.42
NRAMP1 274 C/T	rs2276631	0.19	3.56
SP 110	rs2114592	0.15	3.85
SP 110	rs3948464	0.13	4.06
IFNg +874 A/T	rs2430561	0.29	3.26
IFNg -1616G	rs2069705	0.49	3.41
IFNg +3234	rs2069718	0.43	3.27
IFNgR1 -56 C/T	rs2234711	0.45	3.31
IL-1a -889 C/T	rs1800587	0.38	3.22
IL-1B +3953 C/T	rs1143634	0.12	4.19
IL-10 -1082 G/A	rs1800896	0.35	3.21
IL-10 -592 A/C	rs1800872	0.42	3.26
MBL codon 57	rs1800451	0.15	3.85
VDR Taq1	rs731236	0.26	3.31
VDR Fok1 A/G	rs10735810	0.23	3.39
MCP-1 -2518 A/G	rs1024611	0.19	3.56
TIRAP C558T	rs7932766	0.17	3.69
P2X7 1513 A/C	rs3751143	0.12	4.19

These calculations assume 80% power. This table includes SNPs that have previously been reported to be associated with tuberculosis.

*Odds ratios greater than this value could have been detected in this study.

There were other limitations of this study. First, of the SNPs tested that had previously been associated with tuberculosis, most pertained to pulmonary rather than extrapulmonary disease. Due to possible differences in the pathophysiology of these two disease manifestations, [7] one might expect that not all of the SNPs tested would be related to the pathogenesis of extrapulmonary tuberculosis. Second, not all genes that are presumably associated with tuberculosis pathogenesis had SNPs included in the GeneChip^® ^Human Mapping 50K Xba Array, so not all such genes could be assessed. Third, we were unable to directly incorporate cytokine response data into the genetic analyses because the methodology used to quantify cytokine responses was not the same in the two immunologic studies.

Additionally, concerns with multiple testing arise when screening such a large number of genetic variants. The results of the first approach are presented without any corrections for multiple testing. Because these variants have been associated with tuberculosis before, each test represents an independent statistical hypothesis. By using MDR and permutation in the second approach, P values were empirically derived based on the total number of tests at each stage.

## Conclusions

The results of this study suggest that an evaluation of the underlying mechanism(s) of the genetic variants in IL-1β +3953, VDR *Fok*1 A/G, and the TLR2 microsatellite--and their role in the pathogenesis of extrapulmonary tuberculosis--is warranted. Comprehensive immunogenetic studies will contribute to our understanding of tuberculosis pathogenesis, and may allow us to identify persons at highest risk of developing tuberculosis.

## Methods

### Study participants

The study population was pooled from two immunologic studies that have been described previously; the inclusion criteria for both studies were similar and are described in detail elsewhere[6,7]. Briefly, patients were identified through the Baltimore City Health Department Eastern Chest Clinic and Nashville Metropolitan Health Department Tuberculosis Clinic. In this case-control study, eligibility criteria for case patients included a history of treated culture-confirmed extrapulmonary tuberculosis, age ≥ 18 years old, and HIV-seronegative status. Extrapulmonary disease was defined as any site outside of the pulmonary parenchyma. Exclusion criteria included serum creatinine >2 mg/dL, use of corticosteroids or other immunosuppressive agents at the time of diagnosis or time of study entry, malignancy, or diabetes mellitus. The criteria for pulmonary tuberculosis control patients included HIV-seronegative adults ≥ 18 years old who had completed treatment for culture-confirmed pulmonary tuberculosis, and had no evidence of extrapulmonary tuberculosis. Positive cultures of sputum, bronchoalveolar lavage, or pulmonary parenchyma were required. Controls with latent *M. tuberculosis *infection were ≥ 18 years old, HIV-seronegative, and had a positive tuberculin skin test (defined as ≥ 10 mm induration after intradermal placement of 5 tuberculin units of PPD) without evidence of active tuberculosis. Participants in this control group were U.S.-born (and therefore not vaccinated with BCG), and were mostly close contacts of tuberculosis cases. Exclusion criteria for both control groups were the same as for the case group. Controls were drawn from the same two clinic populations as cases, and were not related to the cases. Extrapulmonary tuberculosis cases and pulmonary controls completed treatment prior to study entry.

This study was approved by the institutional review boards of the Johns Hopkins Hospital, the Baltimore City Health Department, the National Institutes of Health, Vanderbilt University Medical Center, and the Nashville Metropolitan Health Department. All study participants provided written informed consent.

### Sample Collection

CD4+ lymphocytes were quantified by flow cytometry. DNA was extracted from blood samples using the Puregene DNA Isolation kit (Gentra Systems, Minneapolis, MN) following the manufacturer's protocol. Genomic DNA was stored at -70°C until genotyping. Laboratory personnel were blinded to the case-control status of the specimens.

### SNP Genotyping

As of November 15, 2006, 27 SNPs were identified in the literature as being associated with tuberculosis (Table 2). SNPs not included in the GeneChip^® ^Human Mapping 50K Xba Array (see below) were genotyped using validated TaqMan SNP genotyping assays from Applied Biosystems. For each SNP, 25 ng DNA was used. The polymerase chain reaction (PCR) was performed in an ABI 9700 thermocycler under the following conditions: 95°C for 10 minutes followed by 50 cycles of 92°C for 15 seconds and 60°C for 1 minute. The 384-well plates were read on an ABI 7900HT sequence detection system according to manufacturer's manual. The probes were labeled with FAM or VIC dye at the 5' end and a minor-groove binder and non-fluorescent quencher at the 3' end.

### Microsatellite Genotyping

As of November 15, 2006, 3 microsatellites were identified in the literature as being associated with tuberculosis (Table 2). A 5'(GT)_n _microsatellite associated with NRAMP1, a VNTR associated with interleukin (IL)-1RA, and a GT repeat polymorphism in the toll-like receptor (TLR) 2 gene were all genotyped using fluorescent fragment analysis [16]. All 3 microsatellites were amplified by PCR. The PCR primers and conditions for the 5'(GT)_n _and IL-1RA microsatellites were previously reported[17,10]. The TLR2 microsatellite was amplified using the following conditions: 1 minute at 96°C; 30 cycles of 94°C for 1 minute, 1 minute at 53°C, and 2 minutes at 70°C; and a final elongation period of 10 minutes at 70°C. The primers used to amplify the TLR2 microsatellite were previously published [18]. The forward primers for NRAMP1, IL-1RA and TLR2 microsatellites (5'-ACT CGC ATT AGG CCA ACG AG-3', 5'-CTC AGC AAC ACT CCT AT-3', and 5'-GCA TTG CTG AAT GTA TCA GGG A-3' respectively) were all 5' labeled with FAM purchased from MWG Biotech. Following PCR, the amplicons were electrophoresed on a 3730s DNA analyzer (Applied Biosystems) and analyzed with GeneMapper 4.0 (Applied Biosystems).

### Candidate Gene Genotyping

Twenty six candidate genes were identified based on their potential role in tuberculosis immunopathogenesis and the inclusion of SNPs in these genes in the GeneChip^® ^Human Mapping 50K Xba Array (Affymetrix, Inc., Santa Clara, CA). A total of 661 SNPs in these 26 candidate genes were present (Table 4). Genotyping was performed according to the manufacturer's protocol. Briefly, a complexity reduction process was performed where genomic DNA (250 ng) was digested with *Xba*I, ligated to *Xba*I adaptor (Affymetrix), and amplified by polymerase chain reaction (PCR) using primers specific to the ligated adaptor. Cycling conditions were an initial denaturation of 94°C for 3 minutes followed by 30 cycles of 94°C for 30 seconds, 60°C for 45 seconds, and 68°C for 60 seconds. A final extension of 68°C for 7 minutes concluded the reactions. PCR products were assayed by gel electrophoresis, purified, fragmented to < 250 bp using dilute DNaseI (Affymetrix), biotin end-labeled with terminal deoxynucleotidyl transferase, and hybridized to the 50K Xba Array at 48°C for 16 hours at 60 rpm. The hybridized arrays were washed and stained on Fluidics Station 450 and scanned with the GeneChip Scanner according to the manufacturer's settings (Affymetrix). The arrays were analyzed with software GDAS version 3.0.2 (Affymetrix), which provides rank scores for the probability of particular genotypes at SNP loci. The scores were AA or BB for homozygous alleles and AB for heterozygous alleles, and confidence scores showed the accuracy of the genotype call. Standard procedures and default analysis parameters for individual DNA samples were employed. An internal control run in parallel did not detect any DNA contamination. All procedures were performed using the same lots of reagents.

### Quality Control

Genotype calling was performed using GDAS version 3.0.2 software as noted above. This software uses the Dynamic Models algorithm for genotype calling [19]. All markers with significant (P < 0.05 after Bonferroni correction) departure from Hardy-Weinberg equilibrium were excluded from final analyses. In addition, SNPs in the GeneChip^® ^Human Mapping 50K Xba Array with <90% efficiency (>10% missing data) were excluded from the analysis. Missing data patterns did not deviate from random expectations.

### Statistical Analysis

Clinical and demographic characteristics were compared among the three patient groups (extrapulmonary tuberculosis, pulmonary tuberculosis, and PPD+) using the Kruskal-Wallis test for continuous variables and the chi-squared and Fisher exact tests for categorical variables. Missing observations were individually excluded from all stages of analyses (for both the univariate and MDR analyses (no imputation was performed).

Two populations were assessed for genetic factors associated with extrapulmonary tuberculosis: 1) all individuals regardless of race; 2) only black participants. This stratified analysis was performed to minimize spurious associations due to population stratification [20]. There were not enough individuals to perform analyses stratified by other racial groups. The two populations were further divided into three subgroups for association analyses: a) any tuberculosis vs. PPD+; b) extrapulmonary tuberculosis vs. PPD+; and c) extrapulmonary vs. pulmonary tuberculosis.

**Figure 1:**
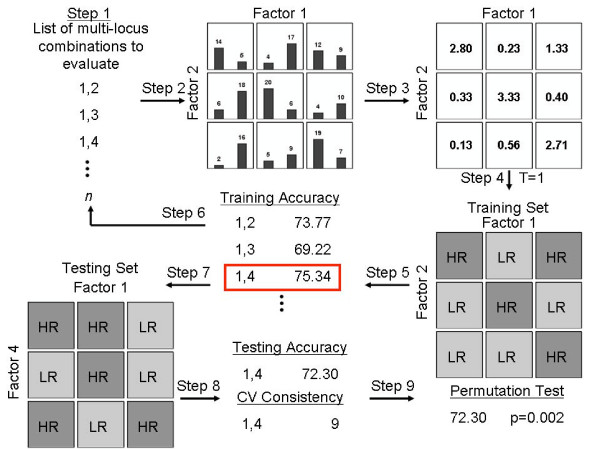
**Summary of the general steps to implement the MDR method, adapted from Ritchie and Motsinger 2005 **[52]. In step one, the exhaustive list of *n *combinations are generated from the pool of all independent variables. In step two, for *k *= 1 to *N*, the combinations are represented in *k*-dimensional space, and the number of responders and non-responders are counted in each multifactor cell. In step three, the ratio of responders to non-responders is calculated within each cell. In step four, each multifactor cell in the *k*-dimensional space is labeled as high-likelihood/high-risk if the ratio of responsive individuals to non-responsive individuals exceeds a threshold and low-likelihood/low-risk if the threshold is not exceeded. In step five the training accuracy is calculated. This is then repeated for each multifactor combination. In step seven, the model with the best training accuracy is selected and evaluated in the test set. In step eight, the testing accuracy of the model is estimated. In step nine a permutation test is conducted to determine the statistical significance of the model(s). Steps 1 through 6 are repeated for each possible cross-validation interval. Bars represent hypothetical distributions of responders (left) and non-responders (right) with each multifactor combination. Dark-shaded cells represent high-likelihood genotype combinations while light-shaded cells represent low-likelihood genotype combinations.

Genetic analyses were performed in two stages (see below). Single locus chi-squared association tests (genotypic tests, with 2 degrees of freedom) (STATA version 9.0; College Station, TX) were performed on variants previously reported to be associated with tuberculosis susceptibility. MDR analyses were performed both on previously reported variants and on variants in candidate genes. MDR was performed to explore potential single-locus associations and gene-gene interactions between variants[21]. Details of the MDR algorithm and implementation are described in the Additional file 1 and Figure 1.

The first stage of the genetic analysis examined variants reported to be associated with tuberculosis susceptibility in previous studies (Table 2). Because each variant represented a distinct statistical hypothesis (since each was being evaluated for replication), no correction for multiple testing was used.

In the second stage of genetic analysis, a nested candidate-gene study was compiled from the Affymetrix Xba genotyping arrays. Only SNPs in genes hypothesized to play a role in tuberculosis pathogenesis were included (see Table 4).

A Linux version of the MDR software was used for data analysis (compiled and benchmarked on a PC with a 600 MHz Pentium-III running Red Hat 2.2.5-15, written in C and compiled with the GNU C compiler). Presently, MDR software is being distributed in a JAVA version with a graphical user interface http://www.epistasis.org/mdr.html.

For both analysis stages MDR was also performed with genetic factors plus CD4+ lymphocytes and body mass index (BMI). This allowed for exploration of both gene-gene and gene-clinical factor interactions.

## Competing interests

The authors declare that they have no competing interests.

## Authors' contributions

AAMR lead the analysis and the writing of the manuscript. PRZA contributed to the design of the study, identification of the genetic polymorphisms to be tested, identification and preparation of the samples to be tested for polymorphisms, and wrote the first draft of the manuscript. NOO contributed to the analysis and the writing of the manuscript. SL oversaw the performance of the genotyping and critically reviewed the manuscript. SMH: contributed to the design of the study and critically reviewed the manuscript. TRS conceived and designed the study, and made significant contributions to the writing of the manuscript. All authors read and approved the final manuscript.

## Pre-publication history

The pre-publication history for this paper can be accessed here:

http://www.biomedcentral.com/1471-2350/11/37/prepub

## Supplementary Material

Additional file 1 **Appendix**. Description of the Multifactor Dimensionality Reduction (MDR) Algorithm and its Implementation. Click here for file
