# Supplementary material for: Polymorphisms in IL-1β, vitamin D receptor Fok1, and Toll-like receptor 2 are associated with extrapulmonary tuberculosis
Source: BMC Med Genet. 2010 Mar 2;11:37. doi: 10.1186/1471-2350-11-37 (PMC2837863; doi:10.1186/1471-2350-11-37)
Supplement: Additional file 1 — Appendix. Description of the Multifactor Dimensionality Reduction (MDR) Algorithm and its Implementation. [file 1471-2350-11-37-S1.DOC]

Figure 1 illustrates the process of the MDR algorithm. Before analysis begins, the dataset is divided into multiple partitions for cross-validation. Cross-validation is an important part of the MDR method, as it tries to identify a model that not only fits the given data, but can also predict based on future, unseen data. Five-fold cross-validation is used, so that 4/5 of the data comprise the training set and the remaining 1/5 of the data comprise the testing set.[1] MDR, then, seeks to find the single-locus or multi-locus model for explaining the outcome (based on a balanced accuracy measure—the arithmetic mean of sensitivity and specificity), based on the available genomic information. [1]

First, an exhaustive list of *n* combinations of genetic loci from the list of all genotypes is created. Next, each of the *n* combinations is arranged in contingency tables in *k*-dimensional space with all possible combinations as individual cells in the table. Then, the number of responders and non-responders for each locus combination are counted. In the third step, the ratio of responders to non-responders within each cell is calculated. Each genotype combination is then labeled as “high likelihood” or “low likelihood” of response based on comparison of the ratio to a threshold. This threshold is the ratio of cases to controls in the data. If the ratio within a multifactor combination is greater than the ratio of cases to control, it is labeled “high likelihood” and if it is less than the ratio, it is labeled as “low likelihood”. This step compresses multidimensional phenotype data into one dimension with two classes.

The high-risk/low-risk distribution for each of the multi-factorial combinations represents the MDR model for a particular combination of multi-locus genotypes. The balanced accuracy for each model is calculated based on the number of individuals within the model that are actually responders in genotype combinations classified as “low likelihood” and the number of individuals that are actually non-responders in the genotype combinations classified as “high likelihood.” Balanced accuracy is implemented into the MDR algorithm to make the process robust to class imbalance (deviation in the ratio of cases to controls from 1.0).[2] The best *k* locus model is selected and the model is evaluated against the testing group, and testing accuracy is calculated. Prediction error, or one minus the testing accuracy, is based on the number of misclassified individuals in the testing set, based on the model developed in the training set. This is repeated for each cross-validation interval (i.e. training set and testing set) and the average training accuracy and testing accuracy are calculated. Among all of the *k*-locus models created, the single model with the highest cross-validation consistency is chosen as the best *k*-locus model. This process is completed for each *k*=1 to *N* loci combinations that are computationally feasible. An optimal *k*-locus model is chosen for each level of *k* considered, so a one-locus model, two-locus model, three-locus model, etc each comprise a set.

Once this set of models is completed, a final model or set of models are chosen. The final model is selected based on maximization of both testing accuracy and cross-validation consistency. Testing accuracy is how well the model predicts risk/disease status in independent testing sets generated through cross-validation, and is calculated as described above. Cross-validation consistency is the number of times a model is identified across the cross-validation sets. For five-fold cross-validation, the consistency can range from one to five. The higher the cross-validation consistency, the stronger the support for the model. When testing accuracy and cross-validation indicate different models, the rule of parsimony is used to choose between them.

Once a best/final model is chosen, permutation testing is used to ascribe significance to the hypothesis generated. Permutation testing involves creating multiple permuted datasets by randomizing the disease status labels (maintaining the ratio of cases to controls in the original data) to create a distribution of a statistic, here testing accuracy, that could be expected simply by chance.[3] One thousand randomized datasets are created and the entire MDR procedure is repeated for each randomized dataset. The best model is extracted for each random data set as described above which generates a distribution of one thousand testing accuracies that could be expected by chance alone. The significance of the final model is determined by comparing the testing accuracy of the final model to the distribution. A p-value is extracted for the model by its location in this empirical distribution. Significance levels are assigned to the final model using the procedure described above and then significant models are reported. It is important to note that MDR analysis produces the most significant model(s), not necessarily every significant model, which is a slightly different goal than the univariate analyses performed for previously associated single nucleotide polymorphisms (SNPs) with logistic regression.

References

(1) Motsinger AA, Ritchie MD. **The effect of reduction in cross-validation intervals on the performance of multifactor dimensionality reduction**. *Genet Epidemiol* 2006 Sep; **30**(6):546-55.

(2) Velez DR, White BC, Motsinger AA, et al. **A balanced accuracy function for epistasis modeling in imbalanced datasets using multifactor dimensionality reduction**. *Genet Epidemiol* 2007 May; **31**(4):306-15.

(3) Good P. Permutation Tests: A Practical Guide to Resampling Methods for Testing Hypotheses. New York: Springer-Verlag, **2000**.
